# Reflex Nervous Troubles from Genito-Urinary Irritation, with Illustrative Cases

**Published:** 1879-10

**Authors:** R. W. Westmoreland

**Affiliations:** Atlanta, Ga.


### REFLEX NERVOUS TROUBLES FROM GENITO-URINARY IRRITATION, WITH ILLUSTRATIVE CASES.

By R. W. WESTMORELAND, M.D., Atlanta, Ga.

*Read before the Medical Association of Georgia, April,* 1879.

My object in presenting this paper, is to contribute some clinical observations upon the nervous disturbance arising from genito-urinary irritation, and not with a view to produce anything original in the way of treatment

Much has been said and written of stricture of the urethra to the extent, not of nausea, but to almost exhaustion of the subject. Whether it arise from congenital defect, or accidental derangement, about the same train of complications result, and about the same urgency obtains for its removal.

In exceptional cases the congeries of symptoms incidental to genito-urinary difficulties derive exaggeration from some peculiar predisposing influences. A case corroborative of this fact I had occasion to observe last year. The patient, a boy about twelve years old, had been suffering a year previously with epilepsy. He had during this time, been treated for enteric irritability as the cause, but with no benefit. I was called in to attend him for an attack of remittent fever, when I gathered the history of his convulsive complaint.

Of immature birth, he had always been very delicate, but had been free from any decided disease up to within three or four months of his first convulsion. A few chills had preceded this paroxysm, when one day having overheated himself romping with some other children, he was seized with a convulsion. This recurred once a month for a few months when appearantly from the influence of an enlarged spleen, and other indications of malarial infection the attacks became more frequent. I treated him for the malarial trouble by administering arsenic, iodine and iron, and for the epilepsy by bromide of potassium and gentian, with the result of lessening the frequency of the spells to some extent. Subsequently the thought suggested itself that the *fons et origo* of the difficulty might be found in genito-urinary disturbance. Accordingly the parts were examined and there appeared a very firm adhesion of the prepuse to the glans, on the right side, extending from the frenum to the middle line on the dorsum. This was only partially broken up, owing, in part, to its firmness, and to the fact that the pain was so great —the patient being incapable of taking anesthetics—that further trials were not permitted.

The local excitement was not accounted sufficient by other physicians who inspected the case, to produce the nervous derangement, yet I am satisfied that could the operation have been completed for removing the adhesion decided benefit would have resulted.

Now, it would appear, as it did to the physicians alluded to in this case, that the effects are too extreme for the cause—the peripheral irritation was too slight for the excessive reflex action that resulted, and that in the production of such inordinate excito-mortor phenomena more manifestation of loca difficulty should have existed, had such been the origin.

It appears in the study of this individual function of the nervous system, that reflex phenomena are in an inverse ratio to sensorial impression, and therefore that the slightest titillation, of the peripheral extremity of an afferent nerve will occasionally produce decided reflex action.

This tendency to sympathetic excitability is not so probable in middle age as in young subjects and in aged persons. The following case, which was reported in the July number of the ATLANTA MEDICAL AND SURGICAL JOURNAL for 1877, will illustrate:

A gentleman fifty odd years of age had been subject to traumatic stricture for twenty-five years. For the latter ten years he was able to urinate only by the most sever exertion. Persevering effort was made to introduce the smallest filliform, without success. Upon external urethrotomy being performed, not only a dense stricture, complicated with a tortuous false passage was discovered, but in addition there was an almond shaped calculus fonnd at the proximal end of the obstruction, lying in a pouch of the uretha. This old gentleman exhibited entire immunity from any reflex complications as a result of the local difficulty, and recovered rapidly. Such a case occurring before puberty, or in a constitution of predisposing susceptibilities, would be very apt to be complicated with decided nervous disturbances.

In stricture or constriction of the meatus, we meet with another source of reflex phenomena.

The term stricture has been objected to when applied to the congenital defect, on the ground that it is not strictly speaking an adventitious deposit, but rather a hyper-formation of the local mucuous membrane during fœtal life. We have instances, however, where closure is attributable to agglutination of the lips by adhesive inflamation, in which case true stricture is closely simulated by the resulting cicatrix. The following is a case in point:

Mr. F.—— aged 21, presented himself complaining of a very acute and excessive urethritis, with warty excresences on the glans penis. He stated that he had contracted gonorrhœa, and had used some "French injection," which he thought aggravated the disease. Denying the possibility of ever having syphilis as a cause of the resulting excresences he gave a supplementary account of having ruptured the frenum sometime previously, and explained in this way the partial closure of the meatus. Upon essaying to use injections for the urethritis, it was found that not even the weakest solution of silver could be borne (1 gr. to f℥). Thereupon the constricted meatus was slit up and steel sounds introduced at regular intervals, while the warts were clipped and touched with lunar costic. In this case there was indigestion, periodic headache, and priapism, which to my mind, were evidences of reflex excitation, which upon removal of the cause disappeared.

In other instances we meet the local trouble uncomplicated with progressive nervous sympathy, though existing as a protracted difficulty. The following is an example:

Mr.——, applied for treatment of a persistent case of urethritis, which had resisted all injections that he had tried. At first I advised a continuance of the plan of injecting, using silver and zinc, but without avail, until I was led to make an examination and found the meatus very much narrowed. I at once opened it up, and the trouble gradually disappeared without other treatment. In this case there was no indication of any reflex trouble though the difficulty had existed sometime.

As to the cause of diversity of nervous impressionability we have to look to the period of development, the era of impubesence on the one hand, and seek an explanation in the nervous peculiarity of that stage. On the other hand, inquiries as to the influences, temperament and education are capable of throwing some light on the subject.

"Hereditary transmission of special susceptibility to irritation, an impressionability of the nervous system and a general weakness of the entire constitution, may be enumerated as predisposing and regulating causes of excito-motor tendencies" (ziem. cyc.) When we have a long maintained chronic inflammation or irritation of the urinary tract, we may expect the nervous system to finally succumb to the exciting cause. Failure in this respect is the exception.

We have other diseases acting in the capacity of peripheral irritants producing perturbation of the ganglionic centres; as calculi, epispadias, hypospadias and phimosis.

Nephritic, calculi create this characteristic disturbance by the irritation produced by the passage of the calculus through the ureters. I once saw a case where the pain from this cause was continuous for two or three days, producing, so the attending physician informed me, during that time a regular epileptic attack.

They may concrete in the bladder, and be sacculate or loose in that viscus, and in either event, be the source of reflex disturbances. Again we find them in the urethra, behind a tight stricture, as in the case presented above, in which location they may act as other foreign bodies, and set up an independent irritation.

When we come to consider the complex process by which the embryonic development is accomplished, and the exact physiological equilibrium that is requisite to the correct arrangement of the infinitesimal particles, the wonder is not that such malformations as epispadias and hypospadias exist, but that they are not more frequent. The mysterious differentiation of cells, commencing in the division of the germinal vesicle, and terminating in the perfection of the respective parts from the epiblast, mesoblast and hypoblast is now imcomprehensible. Yet as such deformities exist and cause not only interruption to the fecundating process, but also decided and diversified forms of nervous disturbance a wide field is presented for surgical ingenuity. Plastic operations promising a cure for these deformities depend for their success upon the distance of the abnormal opening from the glans penis. I have on hand a case in which the abnormal opening is at the root of the frenum, and is so small that great exertion is required to force the smallest stream. Perforating the anterior part, or the glans, with a trocar is suggested, but I have decided on the plan of splitting the part and then uniting the cut edges over a catheter introduced into the bladder. I have seen this plan suggested but once, and that in the Surgical Report of the War. The patient in question is young and shows no sign as yet of any nervous derangement, unless it be in general imperfect or retarded development.

In conclusion, then, I would offer the suggestion that in every instance of spasmodic disturbance occurring during impubesence or at the close of puberty, and the cause is hidden, examine minutely the genito urinary apparatus. If the difficulty is found to originate here, remove the trouble, if possible, at once, for if it is allowed to run on the nervous disturbance may acquire a fixed form that may render the disease almost incurable. The longer such conditions are allowed to remain the more difficult does the cure become. Such is the state of affairs produced by parental solicitude in a case I not long since treated. The little boy, ten years old, had congenital phimosis and preputial adhesion. For about one year he had been having "fits," until they had become as frequent as several during the twenty-four hours. At first the prepuse was slit up, thereby removing the phimosis, and the adhesion, on each side of the glans, broken up. A decided improvement was immediately apparent. The convulsions became much milder and less frequent. It was further proposed in this case to excise the excessively redundant prepuse, and enlarge the slightly constricted meatus. But the parents were reluctant and satisfied themselves with the delusive hope that a cure might accrue from what had already been done. For two or three weeks there was a cessation of the trouble. Recently, however, the father informs me that the paroxysms have returned with increased violence, and in all probability he will now consent to the operation I proposed. Cure in this event may be affected, since excision of the redundant prepuce may permanently remove the adhesions which have recurred since the first operation was performed.

